# Proximal tibiofibular dislocation: a case report and review of literature

**DOI:** 10.1007/s11751-014-0209-8

**Published:** 2014-12-20

**Authors:** R. A. Nieuwe Weme, M. P. Somford, T. Schepers

**Affiliations:** 1 Department of Surgery, Isala klinieken, P.O. box 10400, 8000GK Zwolle, The Netherlands; 2 Department of Orthopaedic Surgery, Medisch Spectrum Twente, Enschede, The Netherlands; 3 Trauma Unit, Department of Surgery, Academic Medical Center, Amsterdam, The Netherlands

**Keywords:** Dislocation, Fibula, Knee, Luxation, Tibia, Tibiofibular joint

## Abstract

An isolated dislocation of the proximal tibiofibular joint is uncommon. The mechanism of this injury is usually sports related. We present a case where initial X-rays did not show the tibiofibular joint dislocation conclusively. It was diagnosed after comparative bilateral AP X-rays of the knees were obtained. A closed reduction was performed and followed by unrestricted mobilization after 1 week of rest. A review of the literature was conducted on PubMed MEDLINE. Thirty cases of isolated acute proximal tibiofibular joint dislocations were identified in a search from 1974. The most common direction of the dislocation was anterolateral, and common causes were sports injury or high velocity accident related. More than 75 % of the cases were successfully treated by closed reduction. Complaints, if any, at the last follow-up (averaging 10 months, range 0–108) were, in the worst cases, pain during sporting activities. We advise comparative knee X-rays if there is a presentation of lateral knee pain after injury and diagnosis is uncertain. Closed reduction is usually successful if a dislocation of the proximal tibiofibular joint is diagnosed. There is no standard for after-care, but early mobilization appears safe if there are no other knee injuries.

## Introduction

The proximal tibiofibular joint facilitates only a little movement with some rotation to accommodate the rotational stress at the ankle joint during dorsiflexion [1]. Isolated dislocation of the proximal tibiofibular joint was first described by Nelaton in 1874 [2]. A dislocation of the proximal tibiofibular joint is uncommon and accounts for <1 % of all knee injuries. It is a mostly sports related [3]. The diagnosis is easily missed on plain AP X-rays of the knee, and bilateral AP X-rays are helpful to identify a proximal tibiofibular dislocation [4]. We describe a case of a proximal anterolateral tibiofibular joint dislocation treated at our facility and include a short review of current literature, published after the classic paper on this condition by Ogden in 1974 [5].

## Case report

**Fig. 1:**
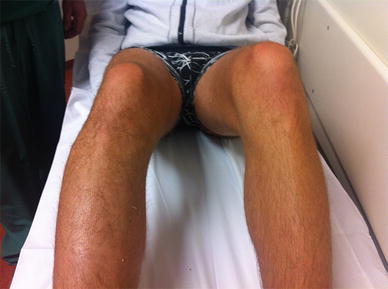
Clinical presentation on the A&E department. The knee could not be further flexed than shown on the picture because of pain. A swelling on the lateral side of the right knee is visible

A 31-year-old male patient presented to our A&E department after sustaining a knee injury during soccer. The exact mechanism could not be elucidated, but the pain started after landing on his right foot after an air challenge. He was unable to bear weight on his right leg. On physical examination, there was a diffuse and discreet swelling on the lateral side of the right knee and local pain on palpation (Fig. 1). Flexion and extension were possible but painful after 110° of flexion on the lateral side of the knee. The knee was stable to stress examination of the ligaments. There was no distal neurovascular deficit.

**Fig. 2:**
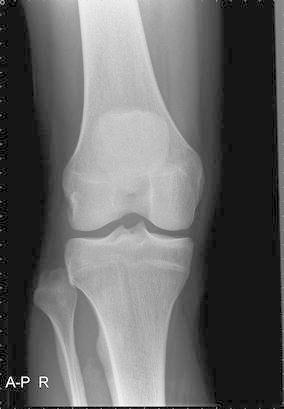
AP X-ray of the right knee. Based on this X-ray, the diagnosis of luxation of the fibula was not made

**Fig. 3:**
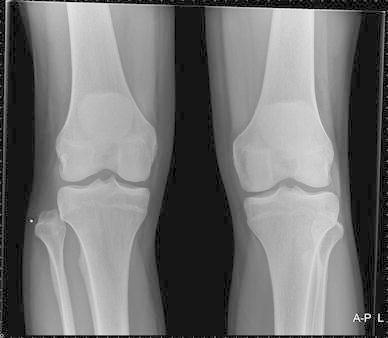
Bilateral AP X-ray of the knees. The aberrant position of the proximal fibula on the right side is evident when compared to the left knee. The direction of the luxation is anterolateral

An AP and lateral X-ray of the knee showed no evident pathology on first assessment (Fig. 2). Diagnosis was made after additional AP X-rays of both knees were obtained. This showed a dislocated proximal tibiofibular joint on the right side (Fig. 3). The fibula was dislocated in an anterolateral direction.

**Fig. 4:**
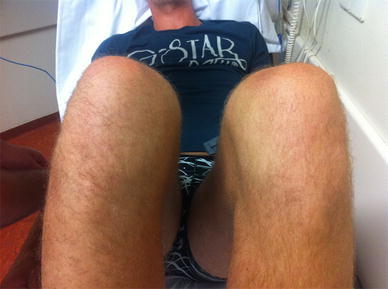
Clinical presentation after closed reduction. The swelling has diminished, and the knee could be fully flexed without pain

**Fig. 5:**
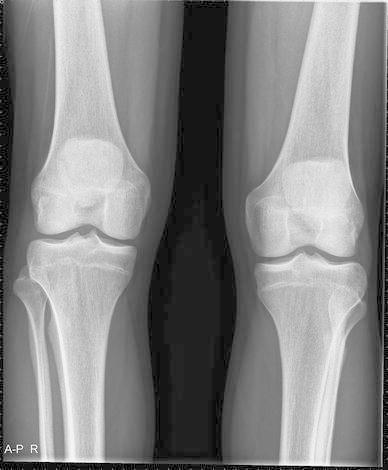
Bilateral post-reduction AP X-ray of the knees showing a reduced fibula on the right side

 A reduction was performed after administering fentanyl and midazolam, with the knee flexed at 90° and the foot in external rotation and eversion, administering direct local pressure using a thumb in the sulcus between the fibula and tibia on the anterior side. Pressure was directed laterally on this location and resulted in a marked pop and instant pain relief for the patient (Fig. 4). Control X-rays showed a reduced proximal tibiofibular joint (Fig. 5).

A three-layer pressure bandage for 1 week and early mobilization was permitted. Further follow-up was uneventful. After 6 weeks, the patient started participating fully in sports. At final follow-up at 6 months, the patient was without any complaints.

## Discussion

The proximal tibiofibular joint is a small joint with minimal movement. The classic paper on proximal tibiofibular dislocation and subluxation by Ogden from 1974 describes four types of instability: atraumatic subluxation, posteromedial dislocation, anterolateral dislocation and superior dislocation. This last type is rare. The most common direction of dislocation is anterolateral in which the mechanism most often involves a violent, twisting motion. This mechanism is commonly seen in various sports, especially during landing after jumping or evading an opponent in team sport [1, 5].

The typical clinical presentation is pain located on the lateral side of the knee after a sports injury. This can be confused easily with a lateral meniscal lesion, especially if guarding because of pain is mistaken for restricted range of motion from a displaced meniscal tear. After establishing the diagnosis, the peroneal nerve should be examined and any changes in sensation noted [1, 5].

In case of an inconclusive diagnosis, plain X-rays of the affected knee in two planes and comparative X-rays of the asymptomatic knee should be taken or, occasionally, an MRI or CT obtained [6].

The reduction should be performed with the knee flexed 90°–110° and externally rotating the foot and applying direct pressure over the fibular head [5, 7].

We searched PubMed MEDLINE for the records of isolated acute proximal tibiofibular joint dislocation since Ogden’s publication in 1974. From the references listed in these published articles, we retrieved other publications on this type of dislocation. Joint subluxation, recurrent dislocation and dislocation concomitant with a tibial fracture were not included in this review. Additionally, spontaneous dislocation or dislocation after a growth disturbance or amputation of the lower leg was not included. This search and review resulted in 30 cases in 21 papers (Table 1) [3, 7–26].

**Table 1:** 30 cases of isolated acute proximal tibiofibular joint dislocation published since 1974

Author	Year	*N*	Cause	Direction	Reduction	After treatment	FU (months)	Complaints
Ahmad [3]	2008	1	Soccer	AL	Open	1-week immobilization	NA	NA
Aladin [9]	2002	1 (bilateral)	Fall from height	AL	Spontaneous	6-week cast non-wb	60	Occasional ache
			Fall during walking	NA	Closed	Tubigrip no restraints	108	Occasional ache
Andersen [10]	1985	3	Motor accident	PM	Spontaneous	6-week cast	36	Pain in sports
			Performing a tackle	AL	Open	3-week non-wb with K-wire	9	Pain after vigorous sports
			Fall running	AL	Open	8-week staple and 3-week cast	7	Pain in strenuous exercise
Buse [11]	1973	1	Landing after jump	AL	Closed	3-week cast	NA	NA
Ellis [12]	2003	1	Snowboarding	NA	Closed	Immediate wb	12	None
Falkenberg [13]	1983	1	Traffic accident	AL	Closed	1-week cast non-wb, 3-week bandage	1	None
Ginnerup [14]	1978	1	Fall on knee	AL	Closed	Full mobilization with support	6	None
Horan [15]	2006	1	Rugby tackle	AL	None	8 days cast, spontaneous reduction on control	NA	NA
Hsieh [16]	2009	1	Three-legged-race	AL	Closed	Protected wb with crutches to full wb in 6 weeks	12	None
Laing [17]	2003	1	Long jump	AL	Closed	6-week mobilization with support bandage	6	None
Levy [18]	2006	1	Twisting injury	AL	Open	12-week non-wb after screw and syndesmosis screws, screw removal 6 months	8	None
Love [19]	1992	1	Fall	AL	Closed	NA	NA	NA
O’Rourke [20]	1982	1	Soccer rotational	AL	Closed	6-week cylinder cast	6	Occasional ache
Pekelharing [21]	2012	1	Soccer rotational	AL	Closed	Functional, 3-week pressure bandage	NA	NA
Petter [22]	2004	1	Hyperflexion	AL	Closed	3-week cast	NA	NA
Pichler [23]	2006	1	Inversion trauma ankle	AL	Closed	Functional	6	None
Rajkumar [24]	2002	1	Landing after jump	AL	Open	3-week cast after PDS pins, 3-week brace	6	None
Schonneman [8]	2012	1	Handball	AL	Open	6-week non-wb after 1 screw, screw removal 16 weeks	12	None
Schuurhuizen [25]	2012	1	Twisting injury	AL	Open	6-week non-wb 2 positioning screws, screw removal 2 months	NA	NA
Thomason [7]	1986	1	Twisting injury	AL	Closed	3-week cast	12	None
Turco [26]	1985	7	Sports	AL	Closed	Elastic bandage and crutches	NA	NA

*N* number of cases, *FU* follow-up, *PM* posteromedial, *AL* anterolateral, *wb* weight bearing, *NA* not available

The most common cause for dislocation of the proximal tibiofibular joint were sports related or from a high-velocity accident. The direction of dislocation reported was almost exclusively anterolateral. In two cases, the direction of dislocation was not specified [9, 12]. In seven cases (23 %), an open reduction was performed [3, 8–10, 18, 24, 25]. All other cases were reduced in a closed manner, three (10 %) of these were spontaneous reductions [9, 10, 15].

The reported after-treatment following a closed reduction was mixed. It ranged from no specific instruction to 6 weeks of immobilization in a long leg cast. The follow-up was 10 months on average (range 0–108). Problems reported on follow-up were occasional aches or pain during sports. But most of the patients did not have residual complaints.

The stated reason for an open reduction was a failed closed reduction, but no explanation was provided as to why the closed reduction had failed. An open reduction was always followed by a fixation; this varied from temporary to definitive fixation, but from the identified papers, we were unable to discern an implant of choice for this fixation.

## Conclusion

We advocate comparative knee X-rays for patients presenting with lateral knee pain after a sports injury for which a diagnosis is not reached from clinical examination or a single set of X-rays. If diagnosed, an attempt at closed reduction is recommended. There is no evidence for the restriction of weight bearing or immobilization after a successful closed reduction. Early movement and weight bearing protected by elbow crutches in the first week is a reasonable after-care protocol, especially if there are no other injuries of the knee diagnosed.
